# A global assessment of cancer genomic alterations in epigenetic mechanisms

**DOI:** 10.1186/1756-8935-7-29

**Published:** 2014-12-04

**Authors:** Muhammad A Shah, Emily L Denton, Cheryl H Arrowsmith, Mathieu Lupien, Matthieu Schapira

**Affiliations:** Structural Genomics Consortium, University of Toronto, MaRS Centre, South Tower, 101 College Street, Toronto, M5G 1L7 ON Canada; Princess Margaret Cancer Centre and Department of Medical Biophysics, University of Toronto, Toronto, M5G 2M9 ON Canada; Department of Pharmacology and Toxicology, University of Toronto, 1 King’s College Circle, Toronto, M5S 1A8 ON Canada; Courant Institute, New York University, 12th floor, 715 Broadway, New York, 10003 USA

**Keywords:** Epigenetics, Pan-cancer, Chromatin, Mutation, Amplification, RNA-seq

## Abstract

**Background:**

The notion that epigenetic mechanisms may be central to cancer initiation and progression is supported by recent next-generation sequencing efforts revealing that genes involved in chromatin-mediated signaling are recurrently mutated in cancer patients.

**Results:**

Here, we analyze mutational and transcriptional profiles from TCGA and the ICGC across a collection 441 chromatin factors and histones. Chromatin factors essential for rapid replication are frequently overexpressed, and those that maintain genome stability frequently mutated. We identify novel mutation hotspots such as K36M in histone H3.1, and uncover a general trend in which transcriptional profiles and somatic mutations in tumor samples favor increased transcriptionally repressive histone methylation, and defective chromatin remodeling.

**Conclusions:**

This unbiased approach confirms previously published data, uncovers novel cancer-associated aberrations targeting epigenetic mechanisms, and justifies continued monitoring of chromatin-related alterations as a class, as more cancer types and distinct cancer stages are represented in cancer genomics data repositories.

**Electronic supplementary material:**

The online version of this article (doi:10.1186/1756-8935-7-29) contains supplementary material, which is available to authorized users.

## Background

Epigenetic control of gene expression dictates cell fate in health and disease, and dysregulation of epigenetic signals is associated with cancer [1, 2]. Two observations support pharmacological targeting of the ‘cancer epigenome’ [1]: (1) some cancer-associated epigenetic aberrations drive cancer initiation or progression; and (2) unlike genetic information, epigenetic states are reversible. Pharmacological agents targeting with little specificity DNA methylation and histone de-acetylation have been approved for the treatment of myelodysplastic syndrome and lymphoma respectively [3, 4], and compounds targeting bromodomain-containing proteins and protein methyltransferases have recently advanced to clinical trials [5, 6]. Cancer associated overexpression, mutation, or aberrant recruitment of chromatin factors (defined here as proteins that participate in the chemical modification of DNA, histones, or control nucleosome occupancy), represent emerging opportunities for cancer therapy. For instance, inhibitors of EZH2 - a histone 3 lysine 27 (H3K27) methyltransferase that is overexpressed in a number of solid tumors and is the site of recurrent gain-of-function mutations in lymphoma - are raising considerable interest as potential anti-cancer agents, and have recently advanced to the clinic [5].

Chromosomal aberrations and altered expression of chromatin factors that are recurrent in specific cancer types have been reported in the literature, some extensively, and recently reviewed [2, 7–9]. Out of the recent compilation of the 58 most frequently mutated genes in cancer [10], we find that 16 are chromatin factors. These aberrations can lead to the deregulation of chromatin patterns controlling hundreds of target genes, as recently reviewed by Plass *et al.*[11]. Pan-cancer analyses of the human genome’s mutational landscape were also recently reported [12–14]. Here, we present a pan-cancer analysis focused on proteins that shape the human epigenome based on the chromosomal and transcriptional landscape of tumor samples from cancer patients available from The Cancer Genome Atlas (TCGA) [15] and the International Cancer Genome Consortium (ICGC) [16]. We took an unbiased approach and focused on cancer types with large numbers of patient samples, but excluding genomes that are extensively rearranged. This systematic and integrated approach identifies many oncogenic aberrations already recorded in the literature, but also uncovers novel alterations recurrently affecting chromatin factors in specific cancer types. Overall our results provide novel insight into the cancer epigenome revealing a tendency toward alterations predicted to result in greater transcriptional repression, decreased transcriptional activation and reduced chromatin remodeling.

## Results

### Study design

Chromatin factors were divided into protein families: protein methyltransferases (PMTs), lysine demethylases (KDMs), histone acetyltranferases (HATs), histone deacetylases (HDACs), DNA methyltransferases (DNMTs), methylcytosine oxidases (TETs), bromodomain containing proteins (BRDs), Royal family of methyl-lysine readers (Kme readers), PHD finger containing proteins (PHDs), and methyl-cytosine binding proteins (MBDs). Some of the members of these protein families are not known to participate in epigenetic signaling, but we followed a target-class approach and included these genes in the analysis. Components of ATP-dependent chromatin remodeling complexes [17] were also added to the analysis as well as histones and their chaperones. Finally, *IDH1* and *IDH2*, two genes that control cellular levels of 2-hydroxyglutarate (an inhibitor of lysine and DNA de-methylases) were included [18], for a total of 441 genes (Additional file 1: Table S1). All pre-processed, validated mutation, and RNAseq expression data affecting these genes in tumor samples were extracted from TCGA (level 3 and 4) and the ICGC (mutation data only - level 3) and grouped by cancer type (Additional file 2: Figure S1). Changes in expression levels and mutation were measured relative to matched normal samples from the same patient. Cancer genomes with unusually high mutation rates (and therefore more passenger mutations) were excluded from the analysis (see Methods section for details). Only cohorts with more than 100 patients passing this filter were considered in order to calculate mutation frequencies. Frequencies of change in gene expression were calculated from cohorts of more than 30 patients. Chromosomal translocations are not included in our analysis. These are directly accessible from literature-based resources such as the Mitelman or COSMIC databases, but we made a deliberate choice here of avoiding data that has been extracted from the literature.

### Uneven deregulation of epigenetic target classes in cancer

As shown in Additional file 2: Figure S1 and Table 1, our analysis retrieved a number of known cancer-associated aberrations in chromatin factors. For instance, EZH2 appears as the most frequently overexpressed protein methyltransferase. We find that this gene is not only overexpressed in breast and prostate cancer, as extensively published [19], but also ranks number 44 and 94 among the most frequently overexpressed genes in liver hepatocellular carcinoma and lung squamous cell carcinoma, respectively. Other examples include recurrent mutations of the chromatin remodeling protein ATRX in lower grade glioblastoma (40% of patient) [20], or DNMT3A and TET2 in acute myeloid leukemia (25% and 8.6% of patients, respectively) [21, 22], mutations of the H3K4 methyltransferase MLL3 in 7.7% of breast cancer patients [23], or mutations of the bromodomain containing protein PBRM1 in 28.5% of kidney renal clear cell carcinoma [24].

**Table 1:** **Most frequently mutated or over/under-expressed chromatin factors in cancer**

Gene	Cancer	Mutated (%)	q value	Gene	Cancer	Overexpressed (%)	Rank (out of 20,544)	Gene	Cancer	Underexpressed (%)	Rank (out of 20,544)
ATRX	LGG	39.8	1.82E-11	HJURP	PRAD	88	3	PRDM16	KIRP	100	14
ARID1A	UCEC	32.3	8.11E-12	UHRF1	BRCA	94.7368	7	PRDM16	KIRC	95.8904	40
PBRM1	KIRC	28.5	1.76E-11	TDRD1	PRAD	84	14	KAT2B	HNSC	82.9268	55
DNMT3A	LAML	25.4	3.96E-12	DPF1	HNSC	87.8049	25	JMJD5	LIHC	88	56
MLL2	LUSC	19.1	6.39E-05	HJURP	BRCA	91.2281	29	TDRD10	LUSC	100	57
ARID1A	STAD	18.1	2.63E-11	HJURP	LUAD	94.7368	31	MECOM	KIRC	95.8904	62
MLL2	HNSC	17.7	1.94E-11	HJURP	LIHC	96	34	L3MBTL4	PRAD	76	73
CHD4	UCEC	13.3	0.000958	ASF1B	BRCA	91.2281	37	ASXL3	THCA	78.7879	81
STK31	SKCM	11.9	0.049702	UHRF1	KIRP	90	37	CECR2	THCA	80.303	83
NSD1	HNSC	10.8	1.89E-11	ORC1	LIHC	94	40	ZCWPW2	HNSC	80.4878	84
SETD2	KIRC	9.8	1.76E-11	EZH2	LIHC	94	44	ZGPAT	LIHC	82	141
TET2	LAML	8.6	2.42E-11	ASF1B	KIRP	90	45	TDRD10	BRCA	85.0877	154
EP300	HNSC	7.8	0.008196	HJURP	LUSC	100	47	MECOM	KIRP	93.3333	162
MLL3	BRCA	7.7	1.95E-11	UHRF1	LUSC	100	68	ACTA1	PRAD	70	194
SMARCA4	LUAD	7.4	2.99E-08	ORC1	LUAD	92.9825	72	CBX7	BRCA	83.3333	218
SETD2	LUAD	7	0.006858	ORC1	LUSC	100	79	TDRD9	THCA	71.2121	261
KDM5C	KIRC	5.8	3.75E-11	ASF1B	LUSC	100	90	TDRD6	BRCA	81.5789	277
ARID1A	LUAD	5.7	0.001823	EZH2	LUSC	100	94	CHD5	PRAD	68	277
SMARCA4	LGG	4.5	0.035502	UHRF1	LUAD	89.4737	101	KIAA1045	BRCA	80.7018	298
KDM5C	LUAD	4.4	0.091808	HIST1H3D	BRCA	85.9649	102	CBX7	LUAD	85.9649	342
ARID1A	BRCA	2.9	0.000117	TCF19	LIHC	88	102	SCML2	THCA	69.697	344
PHF6	LAML	2.5	0.001759	HJURP	KIRP	83.3333	107	L3MBTL4	BRCA	79.8246	346
HIST1H1B	HNSC	2.3	0.084637	CECR2	PRAD	72	107	DPF3	LIHC	72	364
HIST1H3B	BRCA	1.1	9.81E-05	HIST1H3H	BRCA	85.9649	109	CECR2	KIRC	83.5616	388
				UHRF1	LIHC	88	109	PRDM16	LUSC	92	410

Values indicate the frequency of cancer patients where a gene is mutated (non-silent mutations only), where Log2(mRNA tumor/matched control) >1 for overexpression, or < -1 for underexpression. All data were extracted from TCGA and the ICGC. Patient cohorts are greater than 30 for overexpression and 100 for mutations. Hypermutated genomes and other sources of noise were excluded (as detailed in the Methods section). q value is a measure of the *P* value adjusted for false discovery rate. Ranks indicate the number of genes more frequently over- or underexpressed in the human genome.

Our analysis also reveals previously unreported observations. The PRDM sub-group of PMTs is overwhelmingly repressed across multiple cancer types, suggesting tumor suppressor activity, but PRDM12 is among the most overexpressed genes in prostate adenocarcinoma (log2 tumor versus matched control >1 in 66% patients). We also note that PRDM12 is overexpressed in 21 out of 22 colon adenocarcinoma patients (this patient cohort is not presented in our analysis because its size does not meet our criteria for statistical significance (cohort size <30 patients), but this exceptional ratio of 95% is still worth mentioning). The arginine methyltransferase PRMT8 is overexpressed in 68% of thyroid carcinoma (Additional file 2: Figure S1A). Another PMT, MLL2, and the HAT EP300 are found mutated in 18% and 7.8% of head and neck tumors, respectively (Table 1). Interestingly, L3MBTL4, a methyl-lysine reader, is one of the most repressed chromatin factors across all cancer types examined.

**Figure 1:**
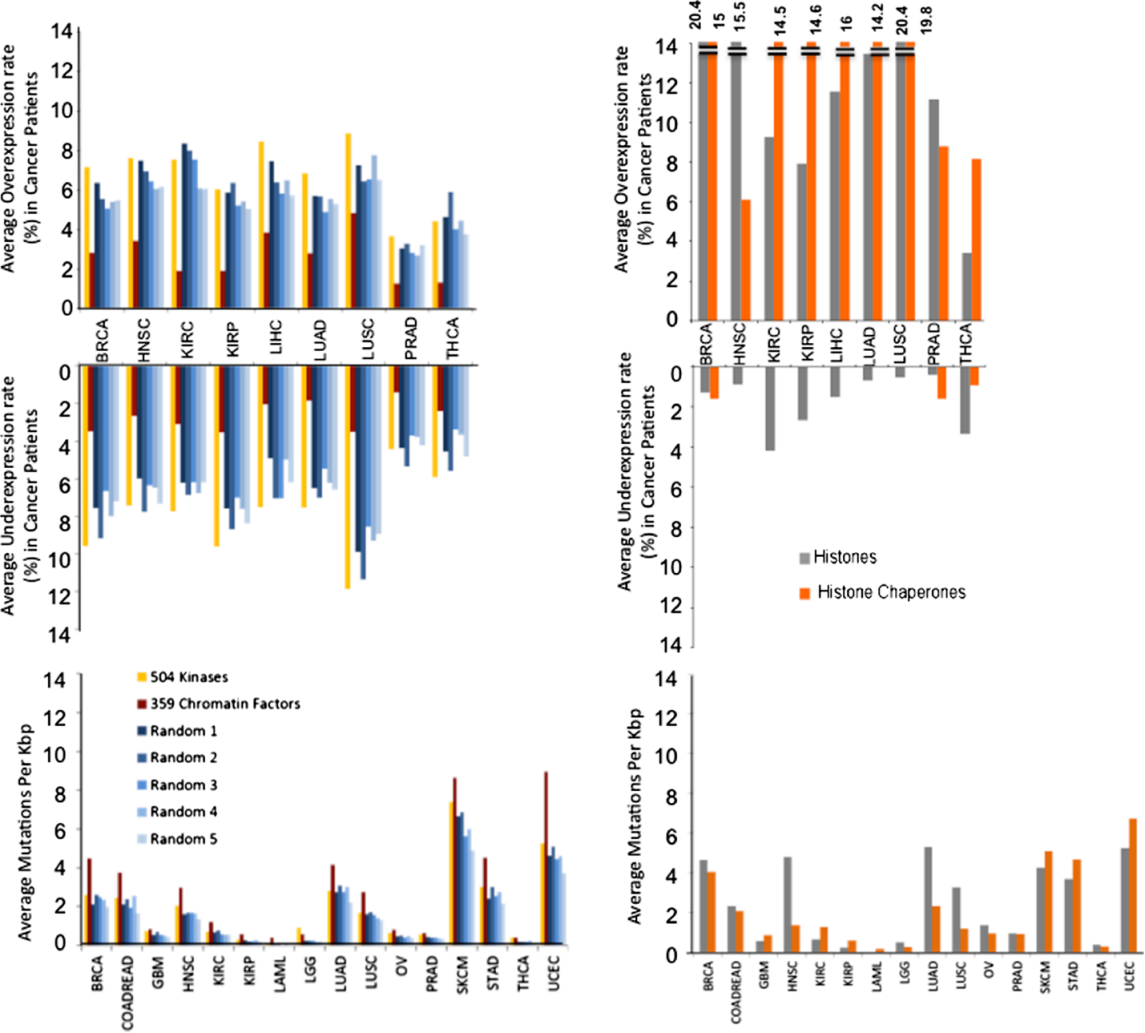
**Average over-/underexpression frequencies and mutation rates of chromatin factor families.** Top: Averages were calculated by summing the frequencies of overexpression or underexpression, or number of mutations per kilobase for each gene and dividing by the total number of genes in the family. The list of 359 chromatin factors is composed of the 441 genes listed in Additional file 1: Table S1 from which histones and histone chaperones were removed. Numbers were also calculated for five independent sets of 359 random genes for comparison. Log2(mRNA tumor/matched control) cut-offs of 2 and -2 were used for over- and underexpression, respectively, for better separation and comparison of transcription profiles.

It has been argued that epigenetic mechanisms are at the heart of cancer biology [9], and it is reasonable to ask whether protein families that control epigenetic signaling represent promising target classes for cancer prevention and treatment. To indirectly address this question, we compared the transcriptional and mutational landscapes of our 441 chromatin genes from which were excluded histones and their chaperones (totaling 359 genes) with those of the human kinome composed of 504 kinases (a validated target class), and five independent sets of 359 random genes (excluding kinases and chromatin factors) (Figure 1). We find that the frequency of altered expression in tumor samples is identical for kinases and random genes, but significantly lower for chromatin factors. Since chromatin factors contribute to regulation of the expression profile of the entire genome, small variations in the expression level of chromatin factors may result in greater changes in the expression level of target genes. Inversely, we find that chromatin factors are more frequently mutated in tumor samples than random genes (Figure 1). Again, the rationale here may be that since chromatin factors control the transcriptional profile of the cancer genome, mutations affecting a single chromatin factor may have a strong impact on the expression of a combination of genes involved in cell fate, survival, or DNA damage response.

Finally, we note that histones and their chaperones are dramatically overexpressed in cancer (Figure 1). This probably reflects strong dependence on histones in a highly replicative cellular environment, such as a tumor. The number of histones frequently overexpressed in cancer could also be accentuated by the fact that histones are clustered within restricted genomic areas that may be co-regulated. Among enzymes, protein methyltransferases are the most frequently overexpressed across the cancer patient cohorts examined (Additional file 3: Figure S2).

Together, these results show that chromatin factors are more frequently mutated in cancer than random genes, but their expression profile is less variable.

### H3K4 and H3K36 methylation are preferentially targeted by mutations

**Figure 2:**
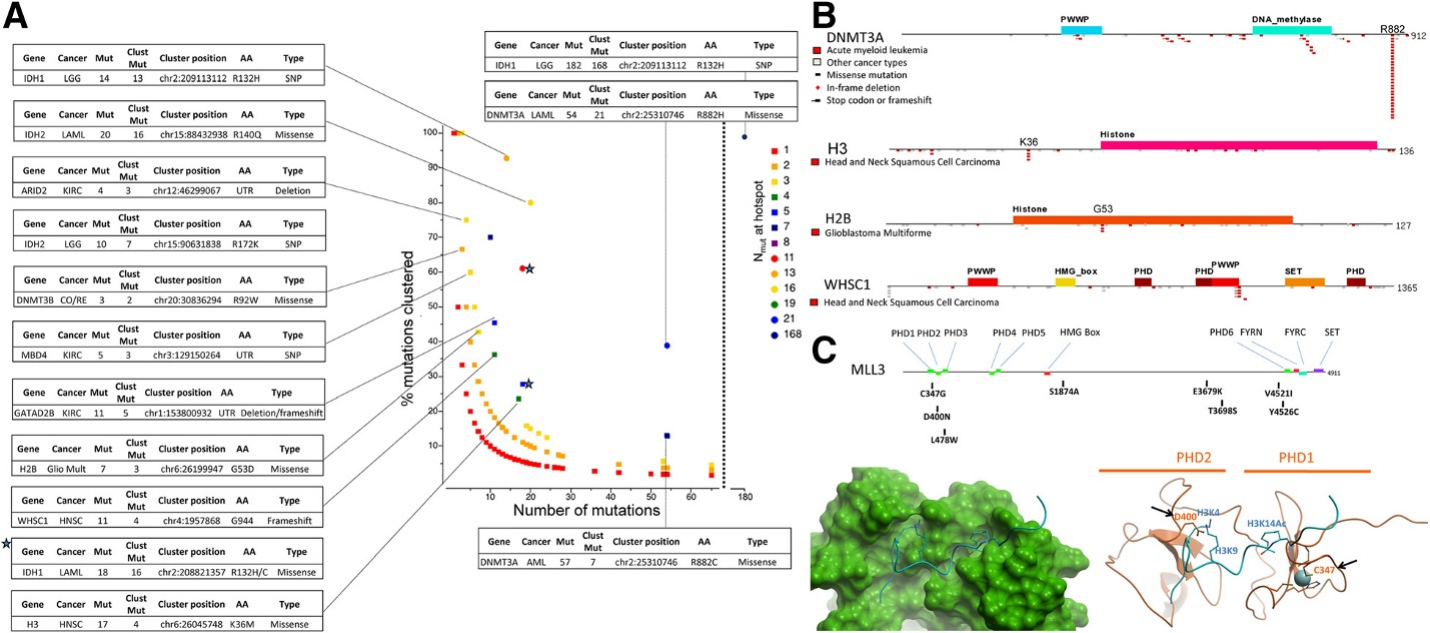
**Mutation frequency and mutation hotspots. (A)** Percent of mutations targeting a conserved residue on a gene (hotspot) are plotted versus the total number of mutations found across the gene in the same cancer type. Color-coding indicates the total number of mutations at the hotspot. **(B)** Mapping of mutation hostpots on the domain architecture of selected proteins. **(C)** Mapping of missense mutations found in colorectal cancer on the domain architecture and 3D structure of MLL3. Three mutations are mapping on an N-terminal cluster of PHD fingers. Two of the mutations that can be mapped on 3D structures are targeting residues that are necessary for histone binding. Hypermutated genomes were excluded from the analysis as detailed in the Methods section.

It has been proposed that site-specific missense mutations that recur across a sizable cohort of cancer patients are indicative of an oncogenic role for the targeted gene, while genes that are frequently mutated at random positions are more likely to act as tumor suppressors [25]. A radiometric rule was suggested in which oncogene candidates should be affected at a recurrent position by at least 20% of missense mutations [25]. Following a similar principle, we searched our 441 chromatin factors for mutation hotspots (Figure 2A,B). As before, this analysis is limited by the depth and breadth of mutational coverage available. For instance, we did not have access to lymphoma data, and failed to retrieve known Y641 mutants that increase the trimethylase activity of EZH2 in this cancer type [26, 27]. However, acute myeloid leukemia (AML) is covered by our analysis, and we did retrieve the well-known mutation hotspot at position R882 of DNMT3A [21]: 21 of the 54 mutations found on this gene in AML patients map at this position (Figure 2A,B). Similarly, the well-known mutation hotspot at R132 of IDH1 recurrent in lower grade glioblastoma (LGG) and AML is observed.

We also identified mutation hotspots that, to our knowledge, have not been previously reported (Figure 2A,B). For instance, genes coding for the histone variant H3.1, are mutated in 17 out of 270 head and neck squamous cell carcinoma samples (HNSC), and four of these mutations replace a lysine with methionine at position 36 (twice in HIST1H3C, once in HIST1H3E and once in HIST1H3I) suggesting that H3K36M is an oncogenic mutation that drives tumor initiation or progression in a fraction of HNSC patients. Interestingly, an H3K27M mutation is observed in 80% of diffuse intrinsic pontine gliomas and 22% of non-brain stem gliomas [28]. The H3K27 methylating PRC2 complex is recruited and trapped by the H3K27M peptide, resulting in an overall decreased methylation of H3K27 at ectopic sites [29]. The authors demonstrated that H3K36M and H3K9M transgenes also decreased overall amounts of H3K36me2,3 and H3K9me2,3, respectively. This suggests that the H3K36M mutation recurrently observed in HNSC patients may result in reduced levels of methylation at H3K36. We also found a H3K36M mutation in a colorectal cancer sample, suggesting that this mechanism may extend to other cancer types. Though statistically significant, we note that the H3K36M mutation rate of 24% out of the 6.2% HNSC samples carrying a mutation at H3.1 remains low. As a comparison, over 40% of cutaneous melanoma samples carry a mutation in BRAF, 90% of which are at the hotspot V600E [30].

Another histone, H2B is mutated in seven out of 377 glioblastoma multiform patients, resulting in a G53D mutant in three cases (in HIST1H2BE, HIST1H2BL and HIST1H2BF) (Figure 2A,B). This mutation places an acidic residue in the minor groove of the DNA wrapped around the histone octamer (Additional file 4: Figure S3), which should destabilize nucleosomal H2B, and possibly nucleosome fluctuation or chromatin architecture.

The PWWP domain is a methyl-lysine reading module that generally binds di- or tri-methylated H3K36. We find that WHSC1, an H3K36 di-methylase that harbors two PWWP domains, is mutated in eight HNSC samples. In four cases, this produces a frameshift insertion at position G944 of the C-terminal PWWP domain (Figure 2B). This results in deletion of the C-terminal helix of the WHSC1 PWWP domain, expected to cap the methyl-lysine binding aromatic cage, and may also cause truncation of the methyltransferase domain of WHSC1, located on a downstream exon. In both cases, alteration of H3K36me2 mediated signaling is expected. We find that the H3K36M mutation and WHSC1 frameshifts are mutually exclusive in HNSC tumor samples. Both aberrations are expected to affect H3K36me2 signaling and may represent alternate pathways to the same molecular endpoint.

While mutation hotspots are expected to reveal oncogenes, tumor suppressors are generally targeted by mutations that are more distributed over the gene in cancer. The tumor-suppressor pattern is predominant in chromatin factors (Figure 2A). We find that the H3K36 trimethylase SETD2 and dimethylase NSD1 are among the top 25 most mutated chromatin factors in kidney, head and neck, and lung carcinoma (Table 1). The H3K4 methyltransferases MLL2 and MLL3 are also among the most mutated, with no apparent mutation hotspot, in lung, head and neck, and breast cancers and are therefore tumor suppressor candidates in these tumors. In total, six of the most mutated genes in various cancer types methylate H3K4 or H3K36 (Additional file 2: Figure S1A, Table 1).

Mutations that are not located at a hotspot appear to be more evenly distributed on target genes, but mapping some of these mutations onto protein structures can reveal ingenious residue targeting. For instance, MLL3 missense mutations are found in eight out of 36 colorectal cancer patients from an ICGC study. Mapping these mutations on the domain architecture of the protein shows that three are located on the N-terminal triple-PHD finger of the protein (Figure 2C - Top). An apo structure of PHD1,2 of MLL3 was solved (PDB code 2YSM), as well as a structure of the tandem PHD domain of DPF3, a close homolog (40% sequence identify), in complex with a histone peptide (PDB code 2KWJ). Superimposing the two structures allows positioning of the histone peptide relative to the MLL3 PHD fingers. Importantly, we observe that D328 makes critical electrostatic interactions with both H3K4 and H3K9 in the DPF3 complex, and is conserved in MLL3 (corresponding residue: D400 - Figure 2C - Bottom). Intriguingly, D400N is one of the three mutations affecting the triple PHD finger of MLL3 in colorectal cancer, and, based on these structural observations, should significantly affect histone binding. A second mutation is C347G. This cysteine is one of the four residues coordinating the Zn atom that holds the first PHD finger together (Figure 2C). The C347G mutation will irremediably affect the structure of this domain, expected to participate in substrate binding. Somatic mutations affecting MLL3 in colorectal cancer seem therefore to target with high precision residues involved in recruiting the enzyme to appropriately marked loci.

Selective targeting of H3K4 and H3K36 methylation by oncogenic mutations was also observed in other studies that are not yet available from TCGA; for instance, mutations in SETD2 and genes affecting H3K36 methylation are recurrent in high-grade gliomas [31]. Together, these results show that H3K36 and H3K4 mediated signaling is highly targeted in cancer via hotspot mutations of oncogenes and random mutation of tumor suppressors.

### Chromatin factors are involved in a brain tumor-specific gene mutation network

**Figure 3:**
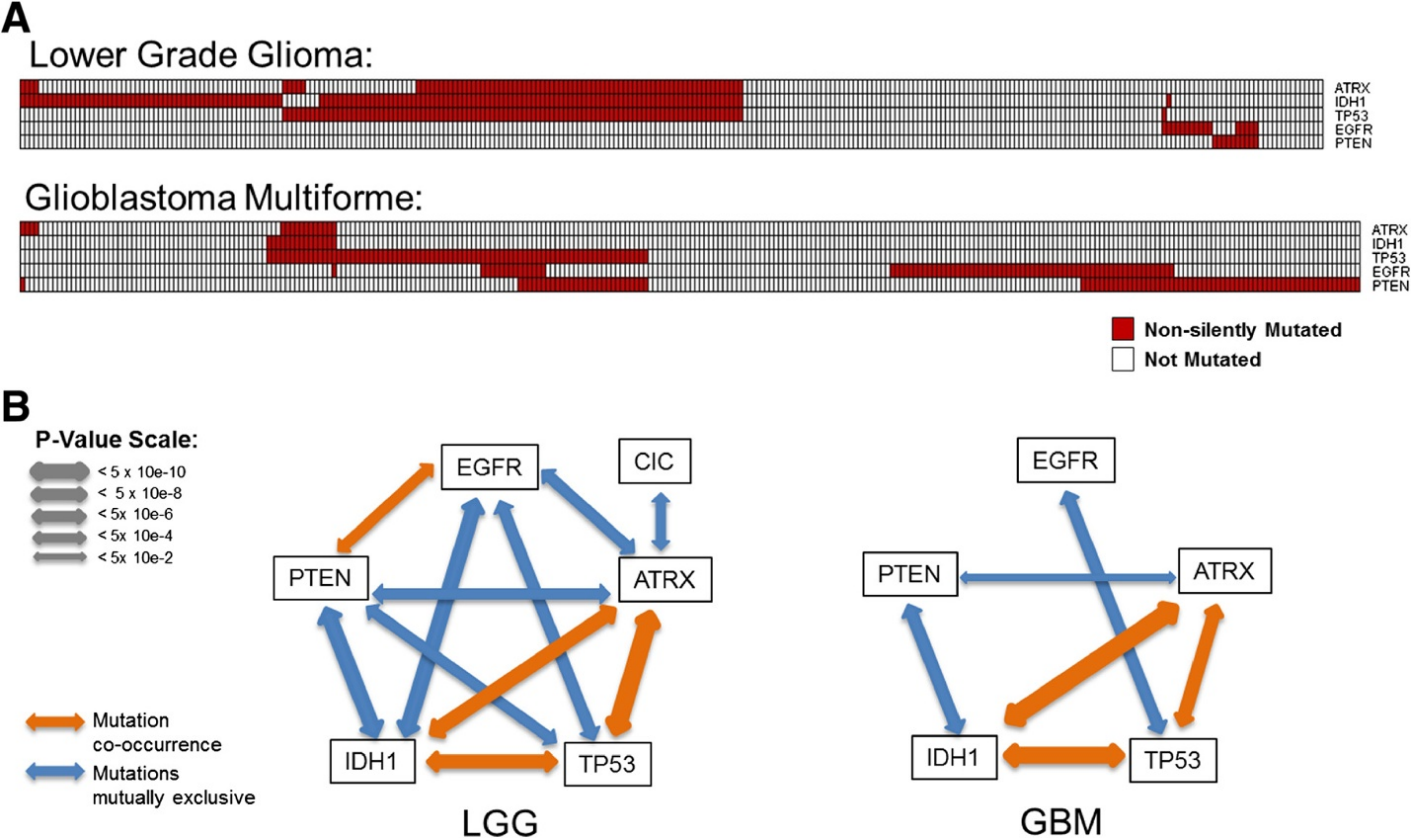
**Mutually exclusive and co-occurring mutations in brain cancer. (A)** Distribution of mutations in ATRX, IDH1, TP53, EGFR, and PTEN across 283 lower grade glioma (LGG) and 288 glioblastoma multiform (GBM) patients. Each column is a patient. Red: mutated; white: not mutated. **(B)** Co-occurrence (orange) and mutual exclusion (blue) patterns suggest that genetic aberrations can target two distinct axes: ATRX/IDH1/TP53 and EGFR/PTEN. LGG and GBM samples with more than 110 and 164 mutated genes, respectively, were excluded from the analysis (cutoffs derived from mutation burden distribution, detailed in the Methods section).

To identify cancer-associated chromatin factor alterations that are either synergistic or redundant, we searched for co-occurring and mutually exclusive mutation patterns, respectively (Additional file 5: Table S2). Co-occurrence or mutual exclusion with non-chromatin factors was also considered. We find that mutations are co-occurring in ATRX, TP53, and IDH1, and that these are mutually exlusive with mutations in PTEN and EGFR in glioblatoma multiform (GBM) and lower grade glioma (LGG) (Figure 3; Additional file 5: Table S2). For example, TP53 is mutated in 50% of all LGG samples, but in 95% of the 80 ATRX-mutated samples.

The mutational landscape of adult and pediatric brain cancer has been extensively analyzed (we did not have access to pediatric data in this work) [32, 33]. Interestingly, it was found that mutations in IDH1, ATRX, or TP53 were recurrent only in glioma-CpG island methylator phenotype-positive tumors (a phenotype probably attributable to the competitive inhibition of TET demethylases, following accumulation of 2-hydroxyglutarate caused by IDH1 mutation), while mutations in EGFR and PTEN were only observed in other tumor subtypes, which is in agreement with the pattern that we observe [32]. An important mutation that is missed in our exome-centric analysis is an upregulating mutation in the promoter of the telomerase reverse transcriptase (TERT), observed in 58% to 84% of primary glioblastomas, suggesting that telomere lengthening plays an important role in tumor growth [34]. Interestingly, ATRX is required for accumulation at telomeres [35], and ATRX mutations promote telomere lengthening and cellular proliferation [36]. Similarly TP53 deficiency favors telomere lengthening [37]. This suggests complementary pressures towards an oncogenic pathway depending on telomere lengthening by mutations co-occurring at ATRX, TP53 and (hypothetically) IDH1 in adult brain tumors where the PTEN/EGFR surface signaling axis is not altered.

Other intriguing observations include a mutual exclusion in lower grade glioma between ATRX and CIC, a transcriptional repressor that may play a role in development of the central nervous system [38], and mutual exclusion in uterine corpus endometrial carcinoma between mutations at TP53 and SWI/SNF remodeling complex protein ARID1A (Additional file 5: Table S2). This is in agreement with a role in maintenance of DNA integrity for both TP53 and the SWI/SNF complex.

### Alteration of chromatin factors involved in replication and genome stability

**Figure 4:**
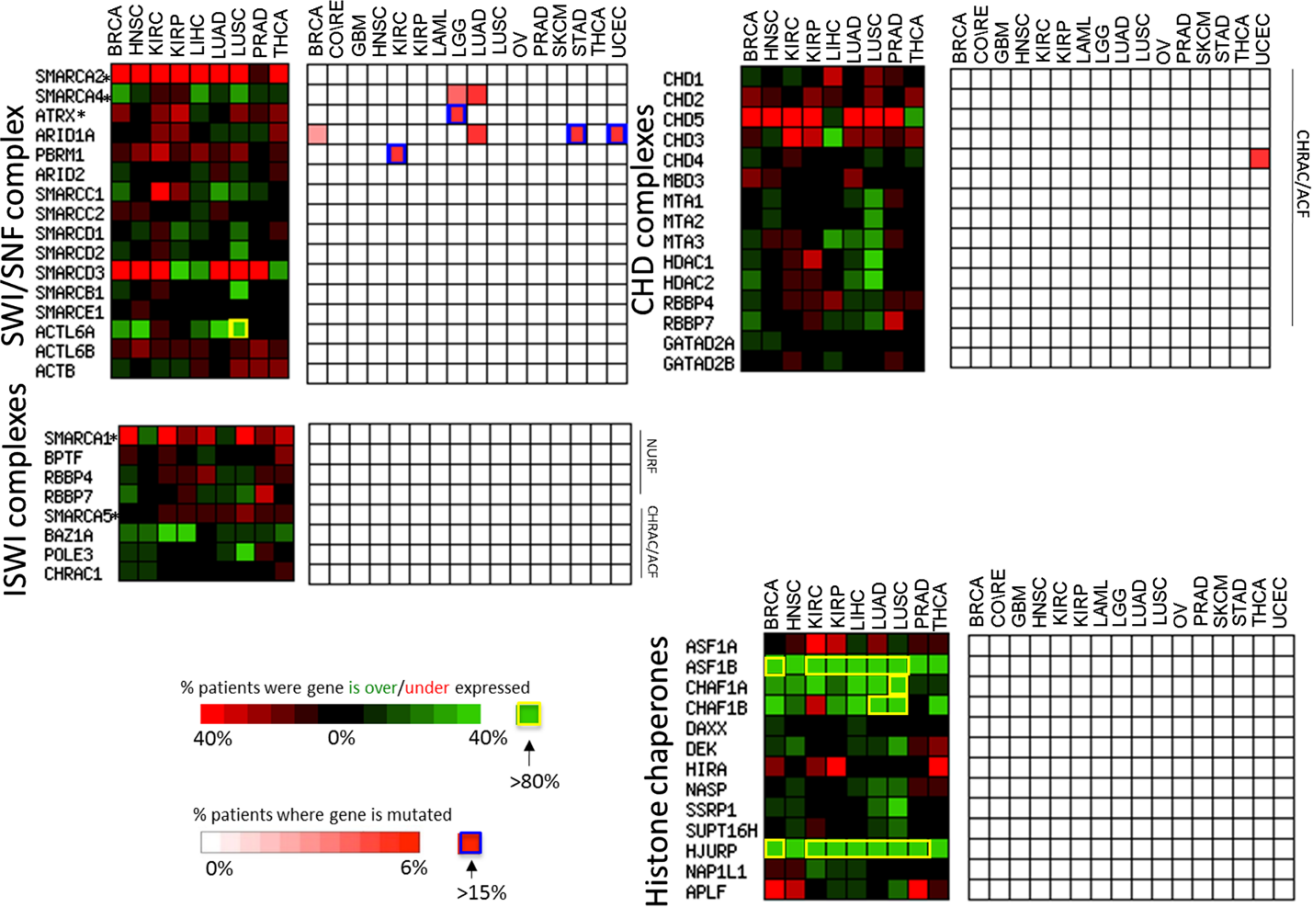
**Alterations of chromatin remodeling complexes and histone chaperones.** Transcription and mutation heatmaps illustrate the frequency of alteration in components of chromatin remodeling complexes and histone chaperones.

We find that some of the changes observed in the cancer epigenome can be associated with a hyperproliferative phenotype, a hallmark of cancer. For instance, histones are twice more frequently overerexpressed than random genes and five to 10 times less frequently underexpressed or mutated in cancer (Figure 1). Additionally, the histone chaperones ASF1B and CHAF1A/B, that are involved in replication-dependent nucleosome assembly [39], are among the most overexpressed histone chaperones, while replication-independent chaperones that maintain nucleosome density and are involved in gene transcription and epigenetic memory, such as DAXX and HIRA [40] are not over-expressed (Figure 4; Additional file 2: Figure S1A,B; Table 1). We also find that the only two proteins known to act as direct links between histone methylation and the DNA replication machinery, ORC1 (that binds to H4K20me3 and recruits the origin of replication complex at replication origins [41]) and UHRF1 (that binds H3K9me3 and recruits DNMT1 to hemi-methylated cytosines [42]), are among the five most frequently overexpressed chromatin factors across all cancer types studied (Additional file 2: Figure S1B).

Another histone chaperone that is significantly overexpressed - actually the most frequently overexpressed chromatin factor in cancer - is HJURP, a chaperone of the histone H3 variant CENP-A, which facilitates aneuploidy and genome instability, another hallmark of cancer [43] (Additional file 2: Figure S1B; Table 1). Expression level of HJURP was previously reported to correlate with glioblastoma cell survival, and was found to be a predictive biomarker for sensitivity to radiotherapy in breast cancer [44, 45].

The DNA repair machinery is an important factor in genome instability, and we find that it is repeatedly targeted through alteration of epigenetic mechanisms in cancer. SETD2 is among the most mutated chromatin factors in kidney renal clear cell and lung adeno carcinomas (Table 1). SETD2 trimethylates H3K36, a mark that regulates DNA mismatch repair through recruitment of the PWWP domain of MSH6 [46]. Additionally, SETD2 was recently shown to act as a guardian of transcriptome integrity by preventing intragenic transcription initiation [47]. The PMT SETMAR includes both methyltransferase and transposase domains, and both domains are essential for double-strand break repair [48]. We find that SETMAR is recurrently underexpressed in tumor samples, including in 54% of HNSC and 78% of kidney renal clear cell carcinoma patients (Additional file 2: Figure S1). ATRX, ARID1A, PBRM1, and SMARCA4 are also among the most frequently mutated chromatin factors in cancer (Table 1; Figure 4), and are all components of the chromatin remodeling complex SWI/SNF, which has been shown to facilitate double-strand break repair [49]. Additionally, ATRX is responsible for the incorporation H3.3 at telomeres, and its mutation can cause alternative telomere lengthening, associated with increased genomic instability [20].

These observations strongly suggest that genetic or transcriptional aberrations targeting chromatin factors in cancer favor replication and contribute to genome instability. We note a potential synergy between these targeted events and other mechanisms that also link epigenetic mechanisms to alteration of DNA maintenance in cancer. These include hypermethylation at DNA promoter regions of genes involved in DNA repair, or direct control of regional mutation rates through chromatin organization [1, 50].

### Gene amplification rarely drive transcriptional alterations of chromatin factors

Cancer genomes generally have large numbers of ‘passenger’ mutations and a small number of driver genetic events. Additionally, cancer-associated overexpression does not necessarily imply disease-relevance. However, when overexpression is caused by a chromosomal aberration, disease-relevance is more likely [51, 52]. To identify candidate drivers affecting epigenetic mechanisms, we looked for correlations between copy number gains and overexpression of chromatin factors in cancer samples compared with matched normal samples [51, 52].

**Figure 5:**
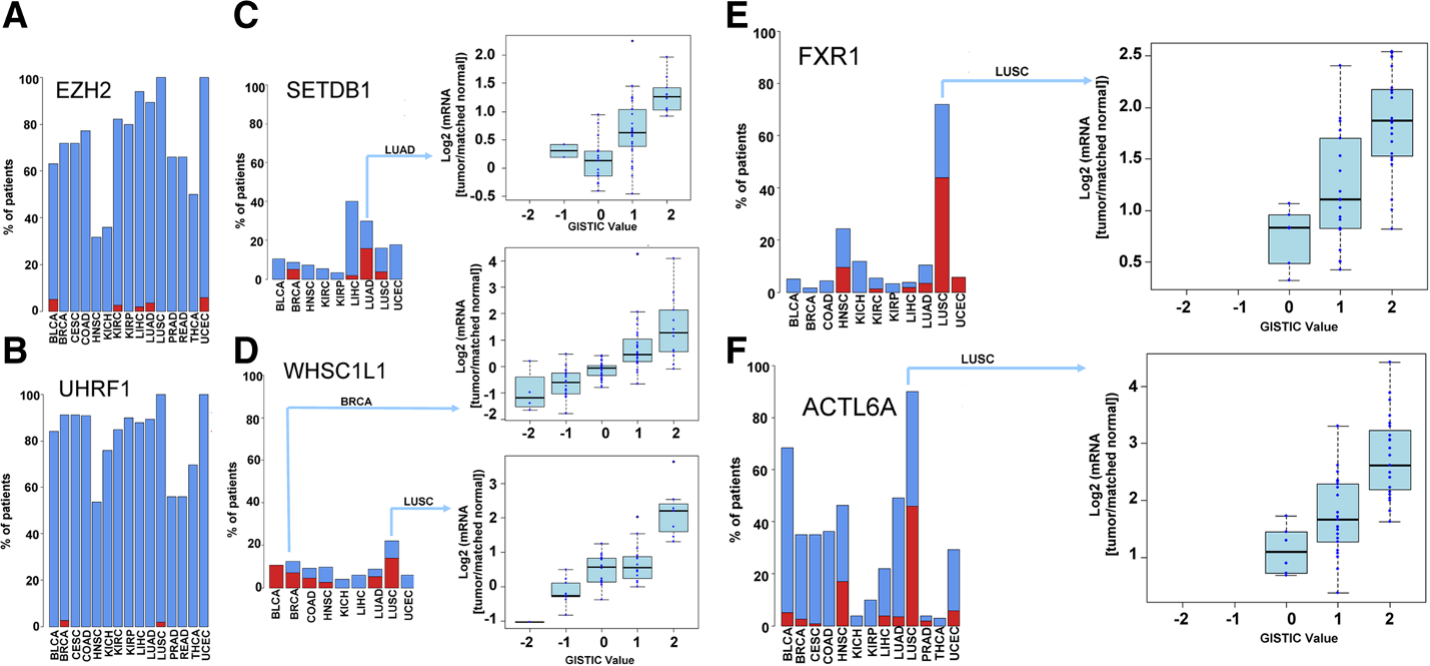
**Correlation between mRNA expression and copy number variation. (A-F)** Recurrence of gene overexpression in tumor samples across different cancer types (blue bars - log2(mRNA(tumor/matched normal)) >1) compared with recurrence of gene amplification (red bars - GISTIC =2 and log2(mRNA(tumor/matched normal)) >1). **(C-F)** Box plots distribution of changes in gene expression levels (log2(mRNA(tumor/matched normal))) in tumor samples grouped by GISTIC values (that is, levels of copy number variation).

We find that in the vast majority of cases, overexpression is not correlated with copy number gain. For instance, EZH2 and UHRF1 are two of the most frequently overexpressed chromatin factors but are rarely amplified in cancer (Figure 5A,B). This comes as no surprise, since multiple factors can affect the transcriptional levels of a gene, such as DNA methylation of promoter or enhancer elements, expression levels of ncRNA, transcription factors or chromatin factors controlling expression of this gene. Nevertheless, we do find that gene overexpression correlates remarkably with gene amplification in a few cases. Clear correlation is observed for the H3K9 trimethylase SETDB1, significantly amplified and overexpressed in 16% of lung adenocarcinoma samples (Figure 5C). Amplification of the *SETDB1* gene in lung cancer was recently shown to contribute to lung tumorigenesis, and shRNA-mediated depletion of SETDB1 in amplified cells reduced tumor growth in a mouse xenograft model [53]. Another example is the H3K36 dimethylase WHSC1L1/NSD3 amplified and overexpressed in 18% and 8% of lung squamous cell and breast invasive carcinomas, respectively (Figure 5D). *WHSC1L1* is in the 8p11.2-p12 amplicon, previously reported in 10% to 15% breast cancers, and associated with poor prognosis [54]. This amplicon includes other genes that may also act as oncogenes, such as *FGFR1*. Interestingly, knockdown of *WHSC1L1* results in profound loss of growth survival of 8p11-12 amplified breast cancer cells, but not control MCF10A cells [55]. These results suggest that amplification of *WHSC1L1* drives cancer in a subset of breast cancer patients. WHSCI1L1 overexpression is even more frequent in lung squamous cell carcinoma and its amplification may also be a driving event in a subset of patients. Among our 441 chromatin factors, the most frequently amplified/overexpressed genes were *ACTL6A*, a component of the SWI/SNF chromatin remodeling complex that is overexpressed and amplified in 53% of lung squamous cell carcinoma samples, and *FXR1*, a gene that codes for a Tudor domain containing protein that is also overexpressed and amplified in 53% of the same tumor type. In both cases, expression levels correlate strongly with copy number gains (Figure 5E,F). Both genes are actually located at the 3q26-29 amplicon, which is prevalent in lung squamous cell carcinoma [56]. Integrative genomic analysis pointed at genes involved in ubiquitylation pathway as candidate drivers, while microarray expression profiles indicated that *FXR1* was one of three genes from the amplicon consistently overexpressed in lung squamous cell carcinoma [57, 58]. Of the close to 250 genes located at the 3q26-29 amplicon, we find that *ACTL6A* and *FXR1* are among the 10 and 30 most frequently overexpressed genes in this cancer type, respectively. Vulnerability of cancer cells to ACTL6A or FXR1 knock-out would be necessary to characterize the role of these genes in lung squamous cell carcinoma.

Together, these results show that overall copy number variation do not appear to drive transcriptional de-regulation of most chromatin factors and are therefore likely to be passenger events in cancer. Nevertheless, in rare cases, recurrent gene amplifications do appear to drive overexpression of a given chromatin factor in tumor samples. Genetic or pharmacologic targeting of these genes will be necessary to further investigate their role in tumor initiation and progression.

## Discussion

Recent landmark next-generation sequencing campaigns of large cancer patient cohorts repeatedly revealed recurrent alterations of genes involved in epigenetic mechanisms [20, 23, 24, 59–61]. The data associated with most of these and other unbiased cancer genomic projects were deposited in TCGA and the ICGC repositories, and made publicly accessible to the scientific community [14, 16]. Here, we took a systematic approach to analyze this aggregated data across a list of 441 genes involved in chromatin-mediated signaling.

**Figure 6:**
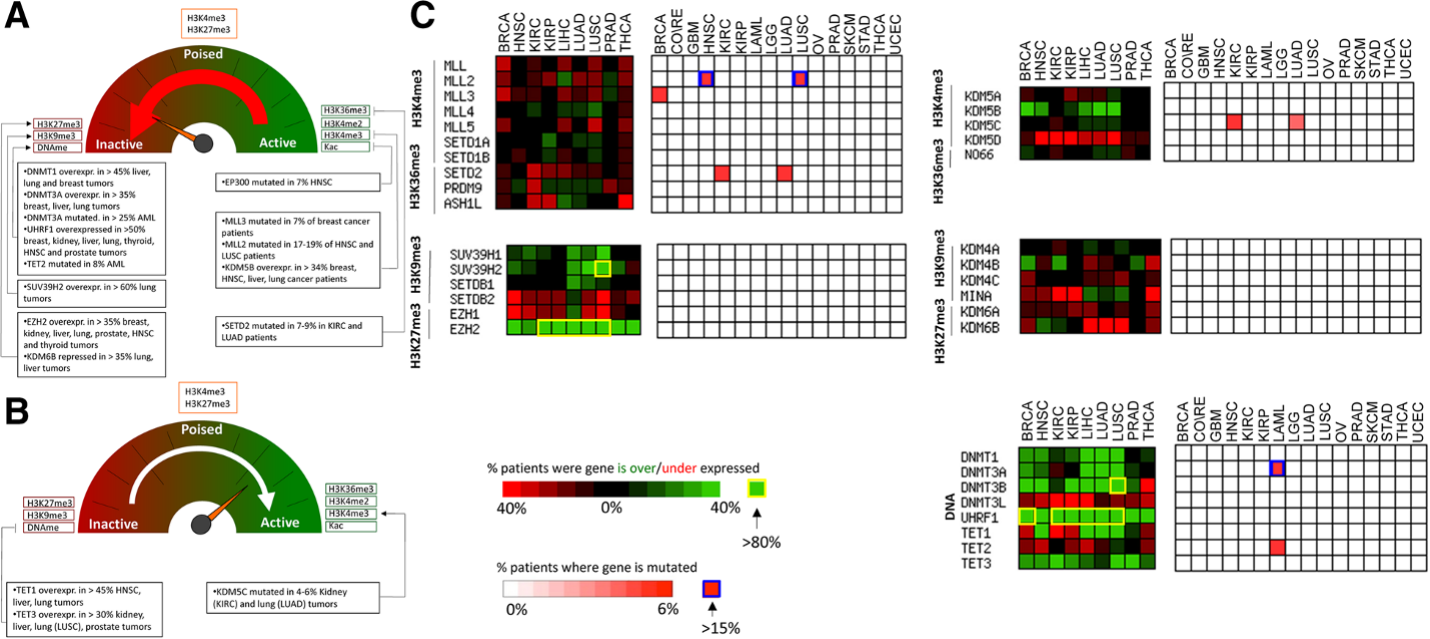
**Mutational and transcriptional alterations associated with activating and repressive epigenetic marks. (A, B)** The epigenetic dashboard reflects the effect of epigenetic states on transcriptional activation status [62]. Most alterations recurrently observed in cancer patients are shifting the epigenetic machinery towards a transcriptionally repressed state (to focus on transcriptional variations of high-amplitude and simplify the list, Log2(mRNA tumor/matched control) cutoffs of 1 and -1 were used for over- and underexpression, respectively). **(C)** Heatmaps are shown for enzymes writing or erasing activating (H3K4me3, H3K36me3) or repressive (H3K9me3, H3K27me3) histone marks, and for enzymes methylating or demethylating DNA as well as UHRF1.

Specific combinations of post-translational modifications of DNA and histones at distinct genomic elements control chromatin compaction, nucleosome occupancy, and gene activation status [62]: histone acetylation and H3K4 di- or tri-methylation at promoters, H3K4 mono-methylation at enhancers and tri-methylation of H3K36 as well as DNA methylation in gene bodies are associated with transcriptionally active genes. Promoters tri-methylated at H3K4 and H3K27 are thought to be in a state that is transcriptionally repressed, but ‘poised’ for rapid activation upon demethylation of H3K37. Finally, tri-methylated H3K9 and methylated DNA at enhancers, or a combination of these two marks with trimethylated H3K27 at promoters, is associated with gene silencing (Figure 6A,B).

Intriguingly, we find that enzymes that deposit histone marks associated with gene activation, such as the H3K4 trimethylases MLL1-4 and SETD1A/B, or the H3K36 trimethylase SETD2 are more often repressed and mutated in cancer (Figure 6C). On the other hand, enzymes that deposit repressive histone marks, such as the H3K9 trimethylases SETDB1 or SUV39H1/2, and the H3K27 trimethylase EZH2 are overexpressed in most cancer types studied. The trend is not as clear for demethylase, but we note that KDM5B, which removes the activating mark H3K4me3, is significantly overexpressed in five of the eight cancer types studied and never repressed, while the H3K27me3 demethylases KDM6A/B are repressed in most cancer types (Figure 6C). Alterations in genes regulating histone methylation appear therefore to be biased towards silencing histone marks. The functional relevance of this observation is unclear. We note that alterations of genes regulating DNA methylation do not follow a similar trend (Figure 6C), and that transcription levels are not repressed in tumor samples when averaging across the whole genome (Additional file 6: Figure S4). In this regard, it is unlikely that a general trend in the control of transcription applies across all tumors, considering the divergence in molecular mechanisms driving different cancer subtypes.

Currently approved epigenetic drugs are DNMT and HDAC inhibitors against myelodysplastic syndrome, acute myeloid leukemia, and lymphoma. With the exceptions of DNMT3B, which is significantly overexpressed in most cancer types studied here, and DNMT3A which is highly mutated in LAML, we do not see notable mutation rates or cancer-associated changes in expression level for DNMTs and HDACs (Additional file 2: Figure S1). We also note that the mode of action of these first generation drugs remains unclear and their toxicity profile mediocre. Some of the emerging epigenetic drugs, such as bromodomain, protein methyltransferase, or IDH1 inhibitors, are targeting patient group with clear oncogenic chromosomal aberrations such as gene fusions at *BRD4* and *MLL1*, or mutations at *IDH1*[6, 63, 64]. Translocations are not included in our analysis, but *IDH1* mutations are high on our chromatin factor mutation landscape (Additional file 2: Figure S1A). Other peaks, such as *ATRX* mutations in lower grade glioma or *ARID1A* mutations in endometrial cancer and stomach adenocarcinoma may represent other points of entry for therapeutic intervention.

## Conclusions

The refined complexity of chromatin as a signaling platform, and its dysregulation in cancer, can only be dissected through systematic identification and functional characterization of all chromatin factors, in specific tissue types, and at specific stages of cancer progression. Here, we apply a reductionist approach to identify general trends associated with protein families or chromatin complexes that are primary determinants of the cancer epigenome. This analysis is restricted by the limited but rapidly growing number of cancer types that are represented at TCGA and the ICGC repositories. It is also limited by restrictions that we imposed to focus on statistically significant patient cohorts and on non-hypermutated genomes (see Methods section for details). It has been proposed that most epigenetic-associated mutations are observed in hematological, in pediatric, or in rare and aggressive variants of solid tumors [9]. It was also noted that, contrary to the general pattern identified here, H3K4me3 and H3K36me3 marks are upregulated during epithelial to mesenchymal transition, an important step in cancer progression [9]. As the volume of cancer genomics data grows, future analysis similar to the one presented here should capture with more accuracy epigenetic transformations underlying distinct types and stages of cancer.

## Methods

### Data access

All raw data analyzed can be accessed and downloaded via the Broad TCGA GDAC Firehose (http://gdac.broadinstitute.org/) or the ICGC data portal (http://dcc.icgc.org/). Somatic mutation, copy number variation, RNASeq gene expression, and DNA methylation data downloaded via TCGA’s Firehose was extracted from the ‘stddata Run’ and is pre-processed. This means the data have been mapped to genes, genomic locations, and a variety of auxiliary data has been added (ex. Amino acid change for mutation data). This type of pre-processed data is referred to as ‘Level 3’ data using TCGA’s nomenclature. Only pre-processed somatic mutation data (version 12) referred to as ‘Simple Mutation’ were downloaded from the ICGC data portal. Further analyzed data, known as Level 4’ , are extracted for somatic mutation and copy number data from the ‘analyses run’. Level 4 data are produced by taking level 3 data and running an algorithm that further isolates statistically significant alterations. For mutation data the algorithm used is MutSigCV [65], while GISTIC 2.0 [66] is used for copy number data.

### Cancer types

In most cases, nomenclature and abbreviations of cancer types used at TCGA and the ICGC were preserved. These are TCGA: BRCA: invasive breast carcinoma, COAD: colon adenocarcinoma, COADREAD: colon and rectum adenocarcinoma, GBM: glioblastoma multiforme, LUAD: lung adenocarcionma, LAML: acute myeloid leukemia, HNSC: head and neck squamous cell carcinoma, KIRC: kidney renal clear cell carcinoma, KIRP: kidney renal papillary cell carcinoma, LGG: lower grade glioma, LUSC: lung squamous cell carcinoma, OV: ovarian serous cystadenocarcinoma, SKCM: skin cutaneous melanoma, STAD: stomach adenocarcinoma, THCA: thyroid carcinoma, LIHC: liver hepatocellular carcinoma, PRAD: prostate adenocarcinoma, and UCEC: uterine corpus endometrioid carcinoma (The Cancer Genome Atlas Network). For ICGC: breast carcinoma, breast cancer, colorectal cancer, glioblastoma multiforme, lung adenocarcinoma, myeloproliferative disorders, chronic lymphocytic leukemia, liver cancer, pediatric brain tumors, and pancreatic cancer. Mutation data downloaded from the TCGA were excluded from the ICGC downloads. In a few cases, distinct patient cohorts from TCGA and the ICGC were affected by similar cancer types. These were merged as follows: breast cancer, breast carcinoma cohorts from ICGC and BRCA cohort from TCGA, colorectal cancer, COADREAD and READ cohorts from TCGA, glioblastoma multiforme cohort from ICGC and GBM cohort from TCGA, lung adenocarcinoma cohort from ICGC and LUAD cohort from TCGA.

### Somatic mutations

Somatic mutations relative to the reference human genome (hg18 for COAD/READ, LAML and OV; hg19 for all other cancer types) are extracted from sequencing data using complex algorithms (which are not discussed here since this pre-processing step is conducted at TCGA and the ICGC) and linked to anonymized patient ID, affected gene/transcript, chromosomal position, and nucleotide/amino acid change. For each patient, the overall number of genes mutated within the tumor sample genome is stored and used to filter out cancer genomes with unusually high number of mutations. This cutoff differs across cancer types based on their mutation level. In order to determine this cutoff, for each cancer type cohort analyzed, the number of mutated genes was plotted across all patients and a value equivalent to the mean +3*standard deviation of the normal distribution was used to set the cutoff (Additional file 7: Figure S5). Any tumor with more mutated genes than the cutoff set for that cancer type was excluded from the analysis when analyzing mutations hotspots and mutation co-occurrence/mutual exclusion, that is, when comparing each individual patient. In other analyses, where the readout is the frequency of mutation, we rely on MutSigCV, which accounts for background mutation rates, gene length, and other source of noise (q value ≤0.1) [65]. No normal distribution, but a continuum of highly mutated genomes were found for lung squamous cell carcinoma and skin cutaneous melanoma, and these two patient cohorts were therefore excluded from our recurrent mutation analysis. Since the frequency of mutation of a single gene is low, cohorts that were less than 100 patients were excluded. Additionally, mutation frequencies that were derived from less than three mutations, and mutations at poly-Q regions were excluded from further analysis to reduce noise levels. The mutation frequencies that we observed for our 1,000 random genes across diverse cancer types differs from that previously published [25]. We attribute this apparent discrepancy due to the fact that level 3 mutation data that we obtain from TCGA and the ICGC are pre-processed to eliminate false-positives. This is in agreement with previous work showing that some cancer types are particularly enriched in false mutation calling. For instance, pre-processing can reduce the number of frequently mutated genes in lung cancer from 450 to 11 [65].

### Co-mutation analysis

Using 2 × 2 contingency tables we produced fisher *P* values, odds ratios, and 95% confidence intervals for each possible pairing of genes present in Additional file 1: Table S1. Two-sided Fisher’s exact test was used to produce *P* values and only those that were ≤0.05 were considered significant. Odds ratios and confidence intervals were produced as previously reported [67]. Odds ratios >1 were considered to imply mutation co-occurrence, while odds ratios <1 implied mutation mutual exclusion. Gene pairs with a confidence interval containing 0 was considered statistically insignificant.

### mRNA expression

TCGA level 3 RNASeq gene expression data were downloaded from the Broad Institute’s Firehose (RNASeq V2 data). Only data from patients with matched tumor and normal samples were used. Cancer types with cohorts under 30 patients were excluded. This threshold is more permissive than the 100 patient cohort used for somatic mutations, as frequencies of transcriptional changes in tumor samples are typically an order of magnitude higher than mutation frequencies. RSEM values are used to quantify mRNA expression levels [68]. A log2 fold change in gene expression is calculated from RSEM values of tumor and matched normal samples as follows:

Frequencies were calculated for each gene and cancer type as the percentage of patients with a log2 fold change greater than 1 for overexpression or lower than -1 for underexpression.

### Average frequencies in over-/underexpression and mutation across protein families

Average frequencies in over-/underexpression and mutation across protein families (Figure 1) were generated for each cancer type by summing frequencies shown in Figure 1 (after changing the Log2 cutoff from 1 to 2 to focus on transcriptional changes of higher amplitude) and dividing by the total number of genes present within the indicated protein family.

### Copy number variation

GISTIC values are used to evaluate copy number variations relative to the reference genome (hg18 for COAD/READ, LAML and OV; hg19 for all other cancer types) [66]. GISTIC values of 1 and 2 indicate moderate and high copy number gains, respectively, while values of -1 and -2 indicate hetero- and homozygous deletions, respectively. All GISTIC copy number data are directly downloaded from TCGA’s Firehose interface (level 4 data). Anonymous patient ID provided by TCGA was used to determine patients where both GISTIC copy number and matched-control RNASeq gene expression data were available. Corresponding patient cohorts with fewer than 15 patients were excluded. These data were used to find correlations between copy number variation and gene expression levels in tumor samples. For interested readers, we made correlations for all cancer types available on the Chromohub website [69].

### Identification of mutation hotspots

Mutation hotspots were defined as aminoacids affected by a minimum of three mutations representing at least 20% of non-silent mutations for that gene in a given cancer type. Highly mutated genomes were ignored, as previously specified.

## Electronic supplementary material

Additional file 1: Table S1: Four hundred and forty-one chromatin factors used in this study and their respective association with epigenetic protein families. (XLSX 30 KB)

Additional file 2: Figure S1: Mutation and transcription heatmaps of chromatin factors. Color codes illustrate the frequency of cancer patients where **(A)** a gene is mutated (non-silent mutations only), **(B)** where Log2(mRNA tumor/matched control) >1 for overexpression, and **(C)** where Log2(mRNA tumor/matched control) <-1 for underexpression. All data were extracted from TCGA and the ICGC. **(D)** Color codes indicate how a gene ranks in the genome based on the frequency with which it is over-/underexpressed in cancer. Patient cohorts are greater than 30 for overexpression and 100 for mutations. Hypermutated genomes and other sources of noise were excluded (detailed in the Methods section). (ZIP 2 MB)

Additional file 3: Figure S2: Average over-/underexpression frequencies and mutation rates of chromatin factor families. Averages were calculated as in Figure 1. (TIFF 3 MB)

Additional file 4: Figure S3: Mapping of the H2B G53D mutation on the nucleosome structure. An aspartate was modeled at position 53 of H2B in the structure of the human nucleosome (PDB code 3AFA). The histone octamer is shown as ribbons (H2B is in cyan). DNA is shown as a mesh colored according to its electrostatic potential (red: electronegative). (TIFF 1 MB)

Additional file 5: Table S2: Co-occurring and mutually exclusive mutations. First tab: all genes. Second tab: focused on ATRX, IDH1, TP53, PTEN, and EGFR in LGG and GBM. (XLSX 3 MB)

Additional file 6: Figure S4: Overall change in expression in tumor samples presenting a genetic or transcriptional aberration affecting a specific chromatin factor. Patient cohorts are groups within box plots where log2(gene expression tumor/matched control) is averaged across the human genome. Cohort sizes for each boxplot are indicated in parenthesis. Light blue: indicated chromatin factor is repressed in tumor samples (log2 <-1). Red: indicated chromatin factor is mutated in tumor samples. (TIFF 449 KB)

Additional file 7: Figure S5: Exclusion of hypermutated genomes was derived for each cancer type from the distribution of the number of mutated genes per cancer patients. The normal distribution section of the curve was used to calculate a cutoff (indicated with a red vertical bar) as the mean of the normal distribution +3 standard deviations. Genomes with more genes mutated than this cutoff were excluded throughout this work. The resulting cutoffs and size of patient cohorts are indicated in the summary table. (PPT 6 MB)
