# Supplementary figures and images for: A global assessment of cancer genomic alterations in epigenetic mechanisms
Source: Epigenetics Chromatin. 2014 Dec 4;7:29. doi: 10.1186/1756-8935-7-29 (PMC4258301; doi:10.1186/1756-8935-7-29)

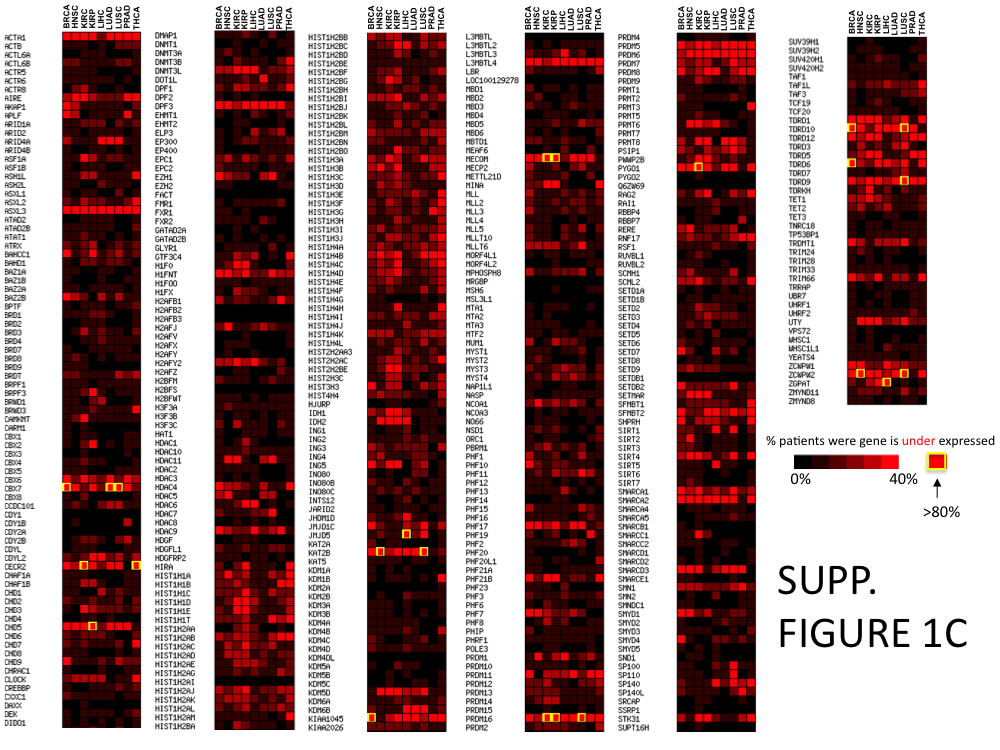

Supplement: Supplementary file 2 — Additional file 2: Figure S1: Mutation and transcription heatmaps of chromatin factors. Color codes illustrate the frequency of cancer patients where (A) a gene is mutated (non-silent mutations only), (B) where Log2(mRNA tumor/matched control) >1 for overexpression, and (C) where Log2(mRNA tumor/matched control) <-1 for underexpression. All data were extracted from TCGA and the ICGC. (D) Color codes indicate how a gene ranks in the genome based on the frequency with which it is over-/underexpressed in cancer. Patient cohorts are greater than 30 for overexpression and 100 for mutations. Hypermutated genomes and other sources of noise were excluded (detailed in the Methods section). (ZIP 2 MB) [file 13072_2014_339_MOESM2_ESM.zip › 4574297139431153_SFig1C.tiff]

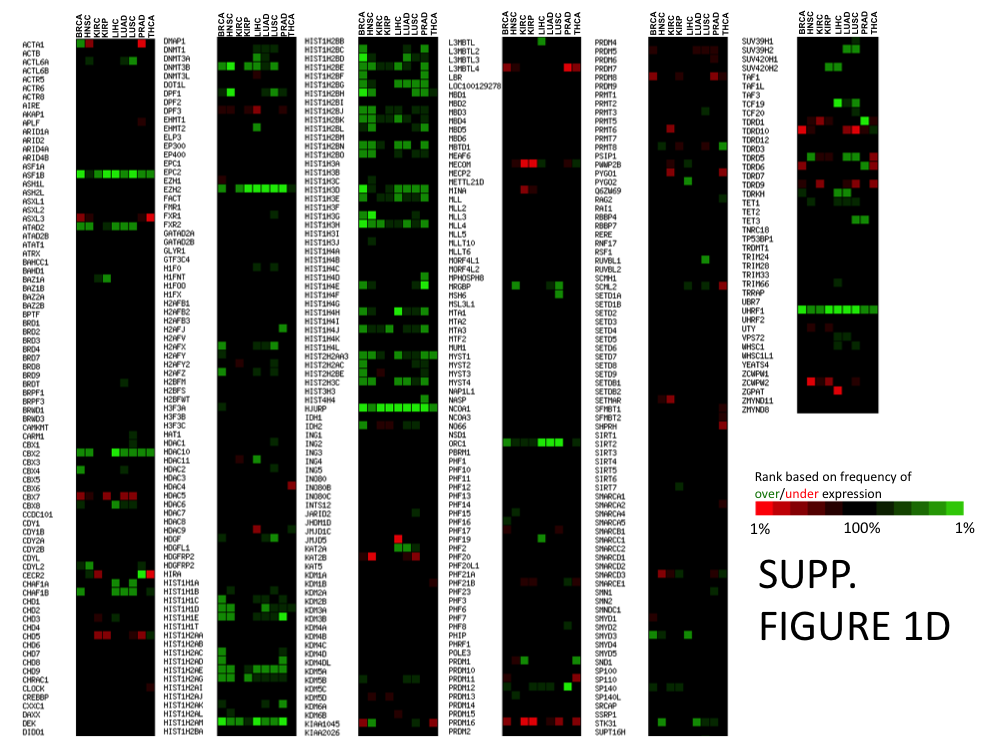

Supplement: Supplementary file 2 — Additional file 2: Figure S1: Mutation and transcription heatmaps of chromatin factors. Color codes illustrate the frequency of cancer patients where (A) a gene is mutated (non-silent mutations only), (B) where Log2(mRNA tumor/matched control) >1 for overexpression, and (C) where Log2(mRNA tumor/matched control) <-1 for underexpression. All data were extracted from TCGA and the ICGC. (D) Color codes indicate how a gene ranks in the genome based on the frequency with which it is over-/underexpressed in cancer. Patient cohorts are greater than 30 for overexpression and 100 for mutations. Hypermutated genomes and other sources of noise were excluded (detailed in the Methods section). (ZIP 2 MB) [file 13072_2014_339_MOESM2_ESM.zip › 4574297139431153_SFig1D.tiff]

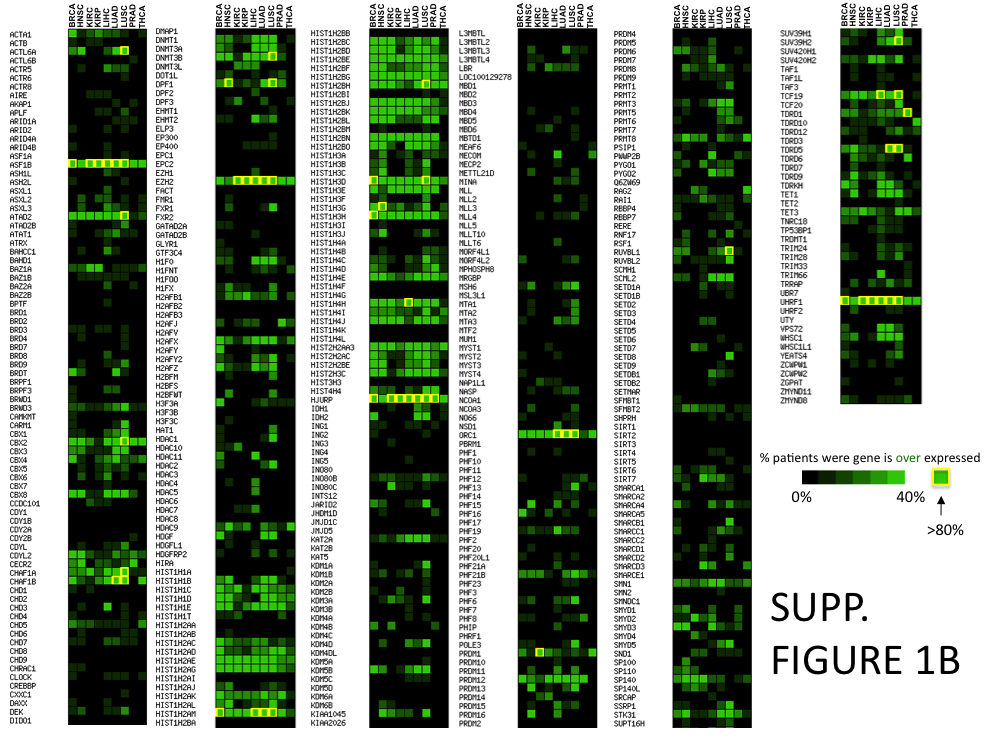

Supplement: Supplementary file 2 — Additional file 2: Figure S1: Mutation and transcription heatmaps of chromatin factors. Color codes illustrate the frequency of cancer patients where (A) a gene is mutated (non-silent mutations only), (B) where Log2(mRNA tumor/matched control) >1 for overexpression, and (C) where Log2(mRNA tumor/matched control) <-1 for underexpression. All data were extracted from TCGA and the ICGC. (D) Color codes indicate how a gene ranks in the genome based on the frequency with which it is over-/underexpressed in cancer. Patient cohorts are greater than 30 for overexpression and 100 for mutations. Hypermutated genomes and other sources of noise were excluded (detailed in the Methods section). (ZIP 2 MB) [file 13072_2014_339_MOESM2_ESM.zip › 4574297139431153_SFig1B.tiff]

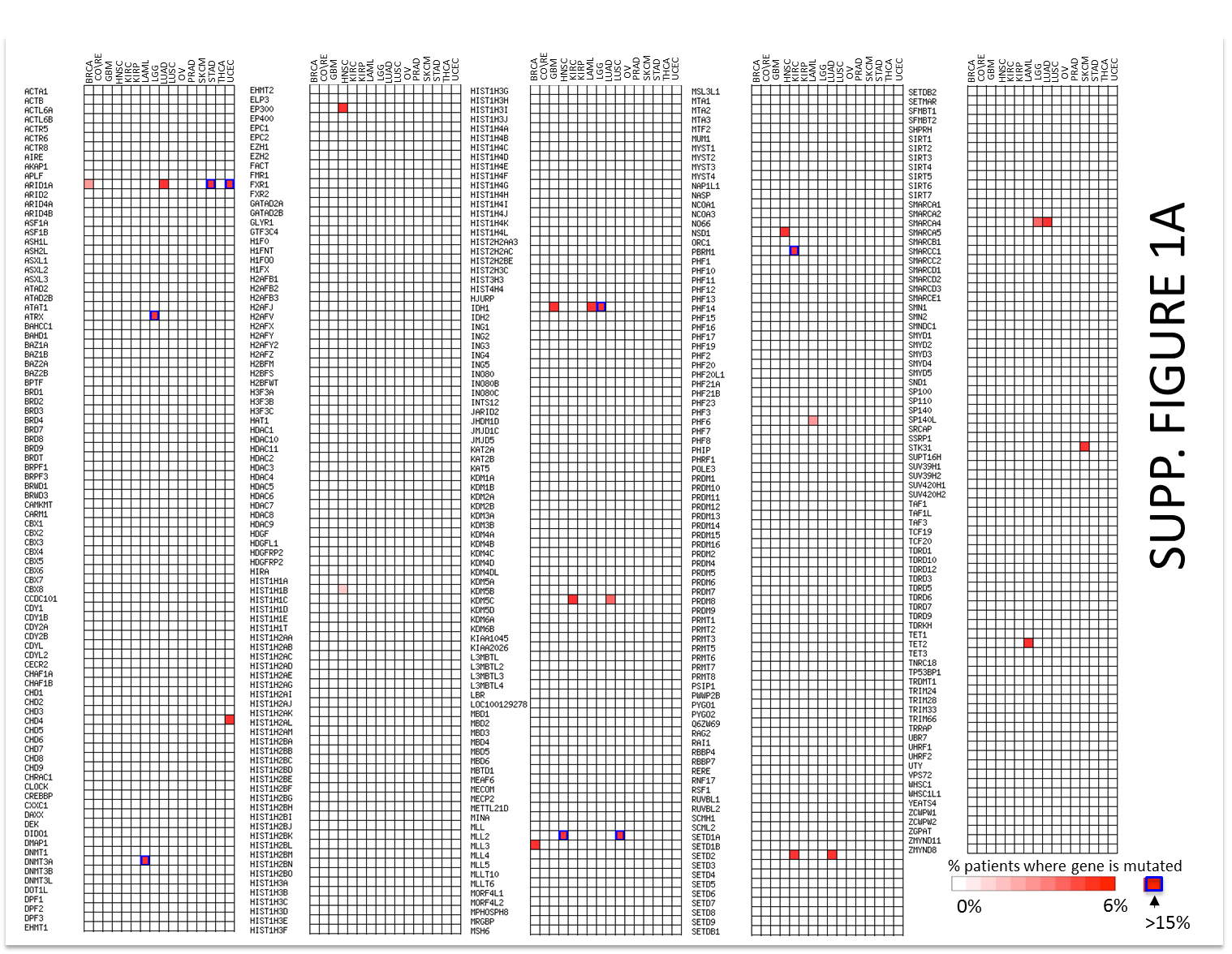

Supplement: Supplementary file 2 — Additional file 2: Figure S1: Mutation and transcription heatmaps of chromatin factors. Color codes illustrate the frequency of cancer patients where (A) a gene is mutated (non-silent mutations only), (B) where Log2(mRNA tumor/matched control) >1 for overexpression, and (C) where Log2(mRNA tumor/matched control) <-1 for underexpression. All data were extracted from TCGA and the ICGC. (D) Color codes indicate how a gene ranks in the genome based on the frequency with which it is over-/underexpressed in cancer. Patient cohorts are greater than 30 for overexpression and 100 for mutations. Hypermutated genomes and other sources of noise were excluded (detailed in the Methods section). (ZIP 2 MB) [file 13072_2014_339_MOESM2_ESM.zip › 4574297139431153_SFig1A.tiff]

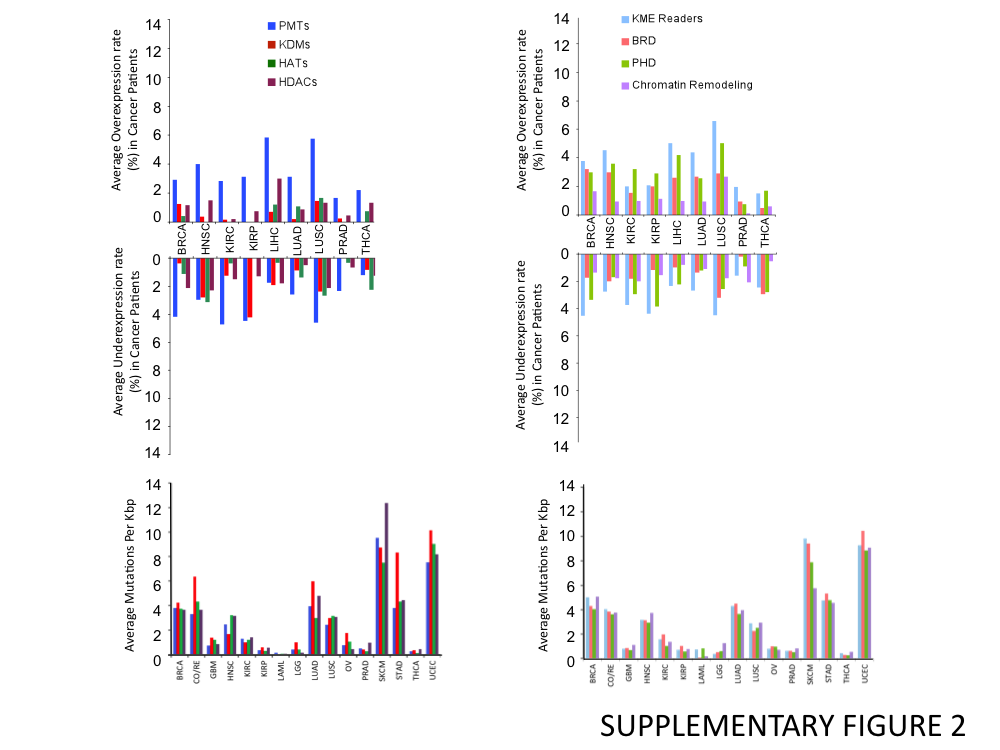

Supplement: Supplementary file 3 — Additional file 3: Figure S2: Average over-/underexpression frequencies and mutation rates of chromatin factor families. Averages were calculated as in Figure 1. (TIFF 3 MB) [file 13072_2014_339_MOESM3_ESM.tiff]

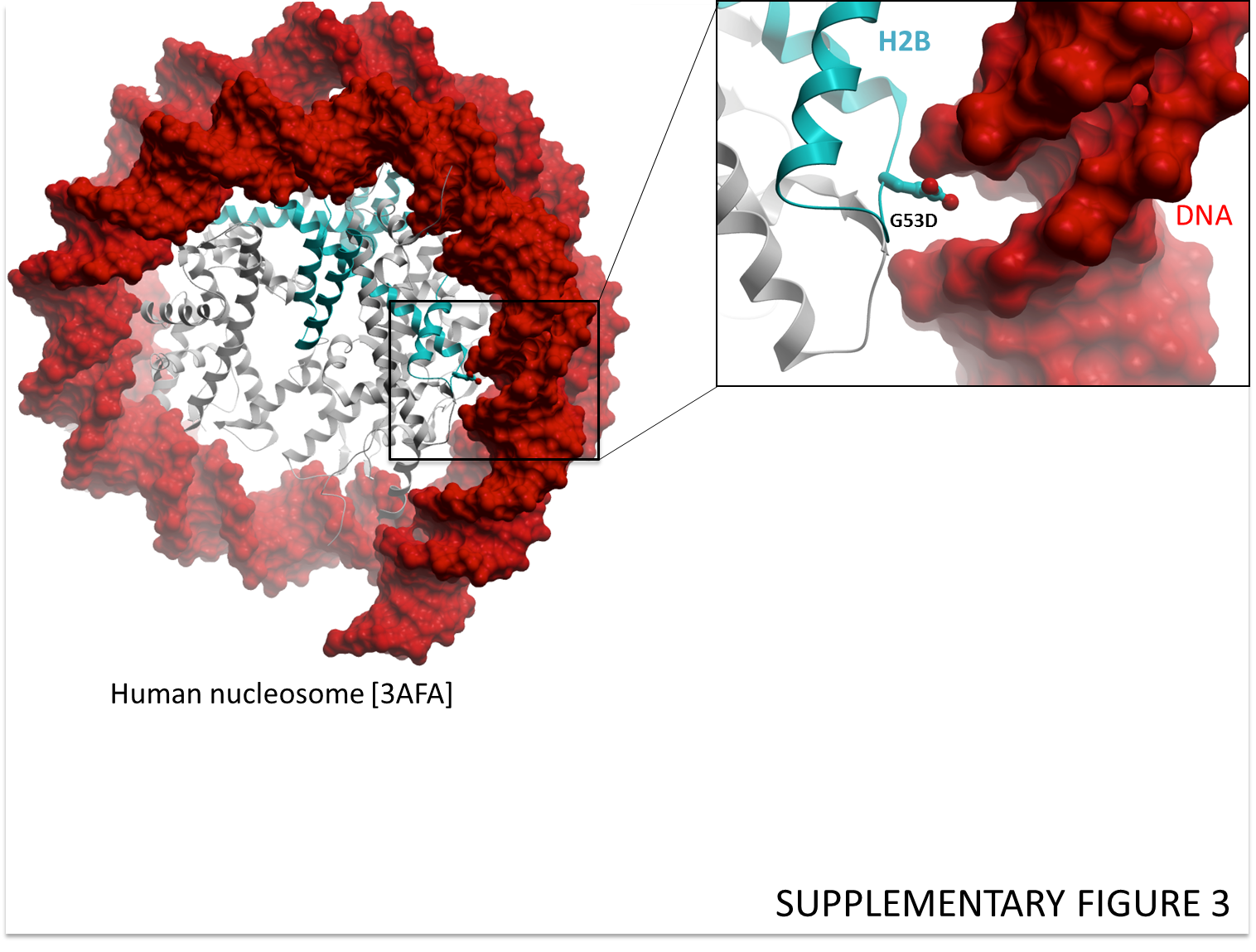

Supplement: Supplementary file 4 — Additional file 4: Figure S3: Mapping of the H2B G53D mutation on the nucleosome structure. An aspartate was modeled at position 53 of H2B in the structure of the human nucleosome (PDB code 3AFA). The histone octamer is shown as ribbons (H2B is in cyan). DNA is shown as a mesh colored according to its electrostatic potential (red: electronegative). (TIFF 1 MB) [file 13072_2014_339_MOESM4_ESM.tiff]

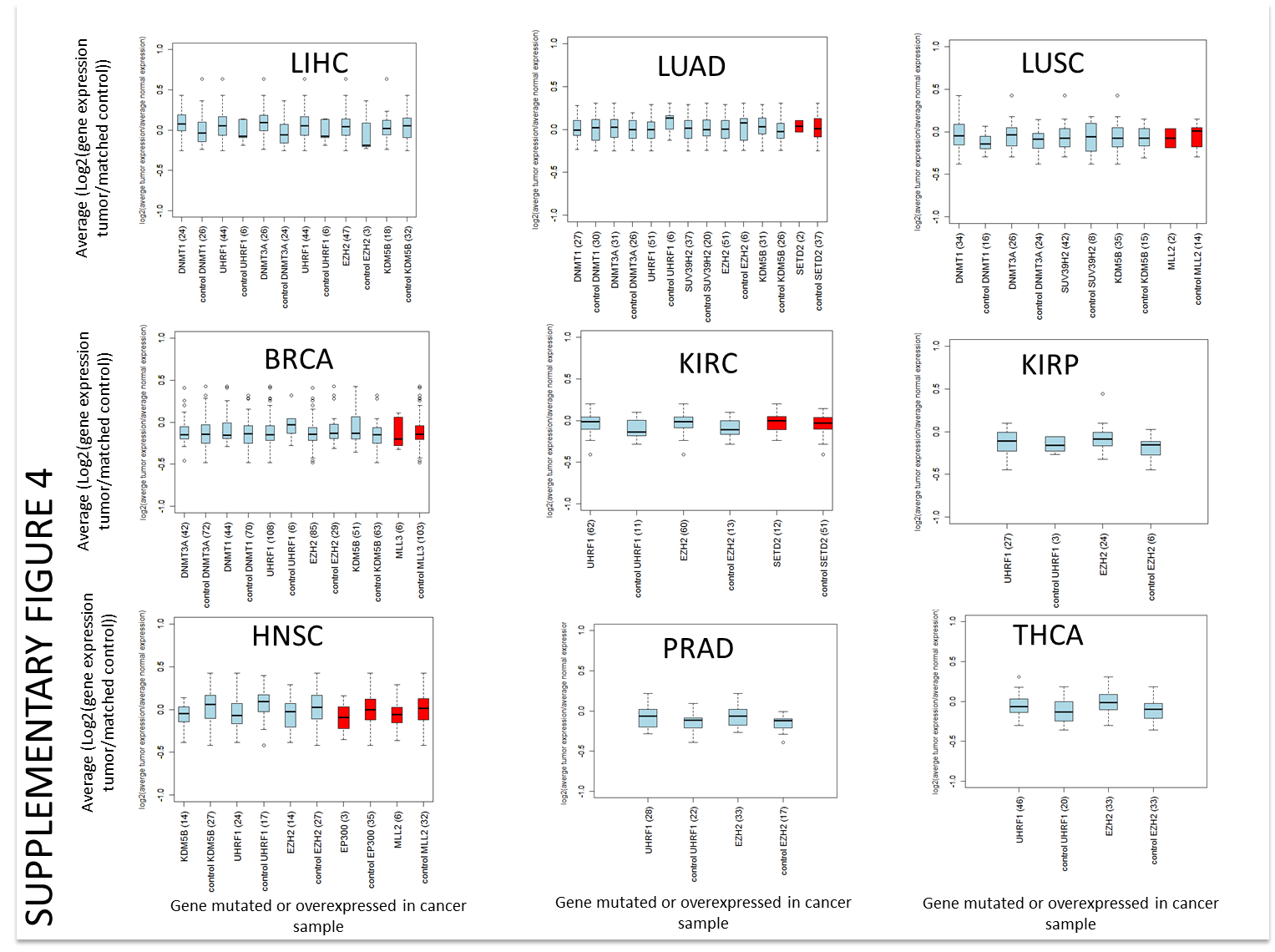

Supplement: Supplementary file 6 — Additional file 6: Figure S4: Overall change in expression in tumor samples presenting a genetic or transcriptional aberration affecting a specific chromatin factor. Patient cohorts are groups within box plots where log2(gene expression tumor/matched control) is averaged across the human genome. Cohort sizes for each boxplot are indicated in parenthesis. Light blue: indicated chromatin factor is repressed in tumor samples (log2 <-1). Red: indicated chromatin factor is mutated in tumor samples. (TIFF 449 KB) [file 13072_2014_339_MOESM6_ESM.tiff]
